# Within the fortress: A specialized parasite is not discriminated against in a social insect society

**DOI:** 10.1371/journal.pone.0193536

**Published:** 2018-02-23

**Authors:** Emilia Solá Gracia, Charissa de Bekker, Ephraim M. Hanks, David P. Hughes

**Affiliations:** 1 Ecology Program, Huck Institutes of Life Sciences, Pennsylvania State University, University Park, Pennsylvania, United States of America; 2 Centre for Infectious Disease Dynamics, Huck Institutes of Life Sciences, Pennsylvania State University, University Park, Pennsylvania, United States of America; 3 Department of Entomology, Pennsylvania State University, University Park, Pennsylvania, United States of America; 4 Department of Biology, Pennsylvania State University, University Park, Pennsylvania, United States of America; 5 Department of Biology, University of Central Florida, Orlando, Florida, United States of America; 6 Department of Statistics, Pennsylvania State University, University Park, Pennsylvania, United States of America; University of Vienna, AUSTRIA

## Abstract

Social insect colonies function cohesively due, in part, to altruistic behaviors performed towards related individuals. These colonies can be affected by parasites in two distinct ways, either at the level of the individual or the entire colony. As such, colonies of social insects can experience conflict with infected individuals reducing the cohesiveness that typifies them. Parasites of social insects therefore offer us a framework to study conflicts within social insect colonies in addition to the traditionally viewed conflicts afforded by groups of low genetic relatedness due to multiple mating for example. In our study, we use the behavior manipulating fungal pathogen, *Ophiocordyceps kimflemingiae* (= *unilateralis*) and its host, *Camponotus castaneus*, to ask if colony members are able to detect infected individuals. Such detection would be optimal for the colony since infected workers die near foraging trails where the fungus develops its external structures and releases spores that infect other colony members. To determine if *C*. *castaneus* workers can detect these future threats, we used continuous-time point observations coupled with longer continuous observations to discern any discrimination towards infected individuals. After observing 1,240 hours of video footage we found that infected individuals are not removed from the colony and continuously received food during the course of fungal infection. We also calculated the distances between workers and the nest entrance in a total of 35,691 data points to find infected workers spent more time near the entrance of the nest. Taken together, these results suggest healthy individuals do not detect the parasite inside their nestmates. The colony’s inability to detect infected individuals allows *O*. *kimflemingiae* to develop within the colony, while receiving food and protection from natural enemies, which could damage or kill its ant host before the parasite has completed its development.

## Background

Cooperation is a major theme in biological organization as different units, from cells to individuals, come together to form a whole which is greater than the sum of its parts [1]. Social insect societies are considered to be paragons of cooperative behavior where individual units (i.e. workers) forgo direct fitness to increase the reproductive output of other individuals (i.e. queens and males). Such altruism is evolutionarily stable because colonies of social insects (i.e. ants, termites, wasps and bees) are composed of kin groups [2–4]. However, such an altruistic system is inherently susceptible to disease and parasitism [5].

Specialized parasites which usurp the bodies of ant workers can be considered as a source of conflict, since the colony’s resources are directed to the parasite’s growth and not directed towards the colony’s fitness. Examples of such specialized ant parasites include: fungi (e.g. *Pandora*, *Ophiocordyceps*), trematodes (e.g. *Brachylecithum*, *Dicrocoelium*), cestodes (e.g. *Raillietina*, *Anomotaenia*), nematodes (e.g. *Mermis*, *Tetradonema*), strepsipterans (e.g. *Caenocholax*), dipterans (e.g. *Pseudaceton*, *Styletta*), and hymenopterans (e.g. *Auxopaedeutes*, *Pseudisobrachium*) [5]. It may be reasonable to hypothesize that the colony would detect infected workers, either because the infected individuals represent a source of infection risk (current or future), or because they represent a drain to the colony’s resources (nutrients go to the developing parasite and not to the reproductives of the colony). An alternative hypothesis is that specialized parasites coevolve with their hosts to develop strategies which limit the colony’s ability to detect the parasite’s presence. By avoiding detection, the parasite takes advantage of both the individual host and society that host belongs to without adverse effects on its development. To test these alternative hypotheses, controlled laboratory studies with a specialized, coevolved parasite system are useful as such a system allows for the examination of the possible changes in behavioral dynamics inside of the nest following an infection and help us determine of social conflicts arise.

Species of the fungal endoparasite complex *Ophiocordyceps unilateralis sensu lato* have closely coevolved with their respective ant hosts and form a useful model to test conflicts occurring inside an ant colony. Fungi of this species complex, such as *Ophiocordyceps kimflemingiae* (= *unilateralis*)[6], are known to commonly infected species of ants in the genus *Camponotus* and *Polyrhachis* [7–10]. In this host-parasite system foraging workers become infected after exposure to fungal spores [11]. The spores attach, germinate, and penetrate into the workers’ cuticle to develop within the hosts’ hemolymph [8, 12, 13]. The fungus develops within these workers for approximately 14–21 days [14], during which time the infected ant is inside the nest. Over the course of fungal development the parasite could be taking advantage of the colony’s resources. When the fungus is ready to transmit, it manipulates its host to leave the nest and bite into vegetation [7–9]. The fungus transitions from growing within the ant’s body to growing a stalk from which spores are produced and released onto the forest floor [7, 10, 11]. Many of these infected workers die at the colony’s doorstep, and infect foragers [15]. It would be advantageous for the colony to recognize the infection in order to reduce the chances of future infection to other colony members.

In this study we tested if infected *Camponotus castaneus* workers: 1) are attacked by nestmates, 2) spend more or less of their time socially exchanging food (i.e. trophallaxis), and 3) are spatially separated from other colony members. We hypothesized that *O*. *kimflemingiae* infected workers are recognized by colony members, and treated aggressively. We expected this aggression to lead infected workers to spend less time in trophallaxis and become isolated inside of the nest. These findings would demonstrate that parasites could bring rise to social conflict in tight-knit communities.

## Materials and methods

### Stock colony collection and maintenance

With permission of private land owners, we collected *Camponotus castaneus* colonies in Douglas, South Carolina during April and May of 2012. We used three colonies for this experiment. Colony 1, collected April 2012, consisted of unmated reproductives that had not yet left for their nuptial flight, brood, and about 120 workers. Colony 2, collected May 2012, consisted of brood and about 100 workers. Colony 3, collected May 2012, consisted of brood and about 100 workers. None of the colonies we collected had queens as these are difficult to collect in this soil nesting species. Once in the lab (Millennium Science Complex at Pennsylvania State University, 40.8017° N, 77.8601° W) we provided all colonies with water and 10% sugar water *ad libitum*, which we replenished once a week. We also provided dead crickets (supplied by Fluker’s Farms) as a source of protein for the developing brood.

### Injection techniques and mortality observations

In order to ascertain which individuals had *O*. *kimflemingiae* development within their body, we used artificial infection methods. We followed the *O*. *kimflemingiae* infection protocols in a similar fashion as those successfully employed in previous studies [14, 16]. Fungal hyphae from a single fungal colony were placed in a sterile 2 mL tube with two 0.63 cm metal balls (Wheels Manufacturing, Inc.) and 200 μL Grace’s medium (Sigma) freshly supplemented with 10% Fetal Bovine Serum (FBS, PAA Laboratories, Inc.). We lysed the fungal colony tissue using a TissueLyser II (Qiagen) at room temperature for 60 seconds at 30 cycles per second. This process enabled us to obtain small segments of fungal hyphae, which we then used at a mean concentration of 3.9x10^7^±1.1x10^7^ hyphae per mL for injection. We infected workers by injecting 1 μL fungal hyphal solution with a laser-pulled 10 μL micropipette (Drummond) and aspirator tube (Drummond) into the thorax underneath the prothoracic legs. Sham treatments were done in similar fashion using 1 μL Grace’s medium supplemented with 10% FBS without fungal tissue.

We performed daily mortality observations for up to 20 days after injections occurred. Any cadavers collected during the experiment were surface sterilized by placing them in 70% ethanol for 20 seconds. After surface sterilization, we placed each cadaver in a sterile petri dish (100×15 mm dimension) containing a Whatman 541 (70 mm diameter) filter paper moistened with 250 μL of sterile water and incubated them at 28°C. In order to determine if these individuals died due to an *O*. *kimflemingiae* infection we monitored the cadavers for fungal growth once a day. We analyzed our mortality data using a Kaplan-Meier analysis with colony and sub-colony as random effects.

### Treatments and individual identification

From each of the three colonies (colonies 1–3), we established two sub-colonies for a total of 6 sub-colonies in all (depicted in Table 1). Each of the 6 sub-colonies contained a total of 35 adult workers. We partitioned individuals into three groups: untreated individuals (“healthy” treatment, n = 15 workers); individuals injected with *O*. *kimflemingiae* plus media (“infected” treatment, n = 10 workers); and individuals injected with media alone (“sham” treatment, n = 10 workers). In order to follow individuals over time, we marked them with unique dot patterns on their head, thorax, and gaster using Edding^®^, number 751 paint markers.

**Table 1:** Colony and sub-colony use.

Colony	Sub-colony	Aggression	Trophallaxis	Time in nest	Spatial data	Mortality
Colony 1	1	X	X	X	X	X
Colony 1	2		X	X	X	X
Colony 2	1	X	X	X		X
Colony 2	2		X	X	X	X
Colony 3	1	X	X	X	X	X
Colony 3	2		X	X	X	X

We mark (with an “X”) which sub-colonies are used for data collection in each category.

### Within-nest behavioral observations

To observe within-nest behavior, we used a modified GoPro camera (Hero 2 fitted with both an infrared [IR] lens and a 4.6 mm macro lens). We situated the camera on top of the colony chamber, and we recorded for 24 hours except for three daily changes of the memory cards (which took between 5 and 15 seconds per change). We housed our experimental sub-colonies in a wooden chamber ranging from 14.93 to 15.46 cm^2^. Each wooden chamber was placed in an individual 452 cm^3^ arena with a sandy floor, which served as a foraging arena. We kept colonies under a 12:12 day-night light cycle with visible spectrum lights from 0600–1800 and infra-red light for the remainder of the 24 hours. Since ants cannot detect light in the infra-red range, it appears dark to them. We kept the colonies at 24°C and 60% humidity. We gave all experimental sub-colonies water and 10% sugar water *ad libitum*. We began our observations three days after we performed our injections to give the sub-colonies time to settle after the disturbance.

We collected our behavioral data by following focal individuals inside of the nest (scoring aggression: total 585 hours, trophallaxis: 655 hours of observations, and spatial data: collected in an 8 hour time frame). The number of focal individuals we followed for each treatment was determined by the number of infected individuals within the nest on the first day of observation. After counting the number of infected individuals inside the nest, we followed the same number of sham and healthy individuals. Unable to differentiate between paint markings under the infrared lights, we were restricted in making our observations during the daylight hours (0900–1700). In order to reduce observational bias, a single observer (ESG) watched the videos. Each video was watched numerous times, since we followed one focal individual at a time. To calculate the total amount of observation time used in this experiment we added the amount of time each individual was under observation. Furthermore, we collected the data for each observed behavior separately.

We measured aggression towards infected individuals until all individuals injected with fungal tissue left the nest. Since the time of nest departure was variable between sub-colonies the duration of observation time was also different between each sub-colony observed. We performed our observations each day from 0900 to 1700. The days of observation for the first sub-colony of each genetic colony were as follows: colony 1–152 hours, days 3–8 post-injection; colony 2–177 hours, days 3–12 post-injection; and colony 3–256 hours, days 3–11 post-injection. Video was played at 10 times the normal speed and paused or played at normal speed if any abnormal behavior occurred. We considered aggressive behaviors as mandible spreading, gaster bending, and lunging forward. We observed a total of 585 hours of video footage to determine if aggression occurred inside of the nest. The presence of infected individuals inside of the nest can affect other social behaviors occurring within-nest, therefore we also test how social food exchange patterns change over the course of fungal infection.

We followed focal individuals inside of the nest to determine if social food exchange (termed trophallaxis) differs while *O*. *kimflemingiae* developed within the hosts’ bodies. We classified trophallaxis as starting when the labrum and labium (i.e. mouthparts of the maxillo-labial complex) were exposed and distended between the two individuals. The event ended when the mouthparts separated and mandibles closed. We watched the behaviors of one focal individual over the course of the entire video. When we finished collecting the data for one individual we would go back to the beginning of the footage to follow a new focal individual.

We collected data on day 6 post-injection during the daylight hours (0900–1700) from six experimental sub-colonies (three distinct genetic colonies), for a total of 655 hours (calculation made by adding the amount of time each worker was followed inside the nest) of observation amongst all the colonies. We chose day 6 post-injection because previous dissections of infected ants revealed that the fungal development was apparent and active by that time (unpublished data). We followed a total of 89 focal individuals, pooling both sub-colonies of each genetic colony together: colony 1 (10 infected, 9 sham, and 9 healthy), colony 2 (10 infected, 11 sham, and 11 healthy), colony 3 (9 infected, 10 sham, and 10 healthy).

Since individuals are able to freely move between our observation (i.e. nest) and foraging arenas, we needed to take into account any variation in the amount of time spent inside. We accounted for the variation in time spent inside the nest by creating a proportion. The proportion of time spent in trophallaxis is equivalent to total time spent in trophallaxis divided by the total time spent inside the nest. Analyzing this proportion data allows us to make inference about the rates at which ants have trophallaxis. Comparing these rates allows us to explore possible differences in trophallaxis rates between ants in different treatments. In our mixed-effect model we used the square root transformation of the proportion of time spent in trophallaxis as our response variable, while treatments were fixed effects. Our random effects included colony, sub-colony, and ant identification. Since each trophallaxis event requires two individuals the data violates the assumption of independence. We therefore used permutation tests as a conservative approach for analysis. The permutation test procedures are explained in more detained in the “Data analysis” section.

Furthermore we used these data to determine the amount of time focal individuals spent inside of the nest. For analysis we used a mixed-effect model, along with a *post hoc* least means squared pairwise comparison amongst treatments, to determine if treatment had an effect on the amount of time a worker stayed within the nest. In our model we used the log transformed data as our response variable, while treatment was a fixed effect. Furthermore we had colony identification, individual identification, and trial as random effects.

### Spatial data collection and analysis of focal ants inside the nest

Within our study we were also interested in determining if individuals infected with *O*. *kimflemingiae* are spatially isolated from other workers inside the nest. We measured distances between the head-thorax juncture for each focal ant inside the nest and the center of the nest entrance every minute from 0900–1700 on day 6 post-injection (n = 35,691 data points). Each measurement was accomplished by first collecting the x-y coordinates of each focal individual inside the nest. We collected the x-y coordinates using a bespoke Python script which created frames (screenshots) of the video every minute (code available upon request). Then an observer (ESG) would collect the x-y coordinates of each worker by clicking on their location.

Observations were performed using all colonies. The number of focal individuals we followed per colony was as follows: colony 1 (10 infected, 9 sham, and 9 healthy individuals), colony 2 (7 infected, 6 sham, and 6 healthy individuals), and colony 3 (9 infected, 10 sham, and 10 healthy individuals); total n was 26 infected, 25 sham, and 25 healthy individuals. To determine if there are significant differences between our treatments we needed to use a permutation test, coupled with a mixed-effect model.

To determine if treatment had an effect on the proximity to the nest entrance we used a mixed-effect model. The model used treatment as a fixed effect, while colony, sub-colony, and individual identification were random effects. Since the assumption of independence is violated in our spatial data set, one worker can affect the movement of another and two workers cannot occupy the same space, we used a permutation test to perform a conservative analysis. The methodology used for the permutation test can be found in the Data analysis section.

### Data analysis

We analyzed our data sets using the R program version 3.3.0 [17]. The packages we used for analysis included survival, lme4, lsmeans, and also a custom code for our permutation tests [18, 19]. Since trophallaxis is a pair-wise event and two workers cannot occupy the same spatial location at once, the datasets violated the assumption of independence and needed to use a permutation test. The code we created randomly permuted the treatment of each individual, analyzed the results using our mixed-effect models, and stored the parameter values for the 10,000 permutations. We then calculate an empirical p-value for the regression parameter estimates from the original treatment assignments by finding the proportion of the 10,000 permutation regression parameters that have absolute value larger than the regression parameter estimate from the original data.

## Results

### Survival analysis

**Fig 1:**
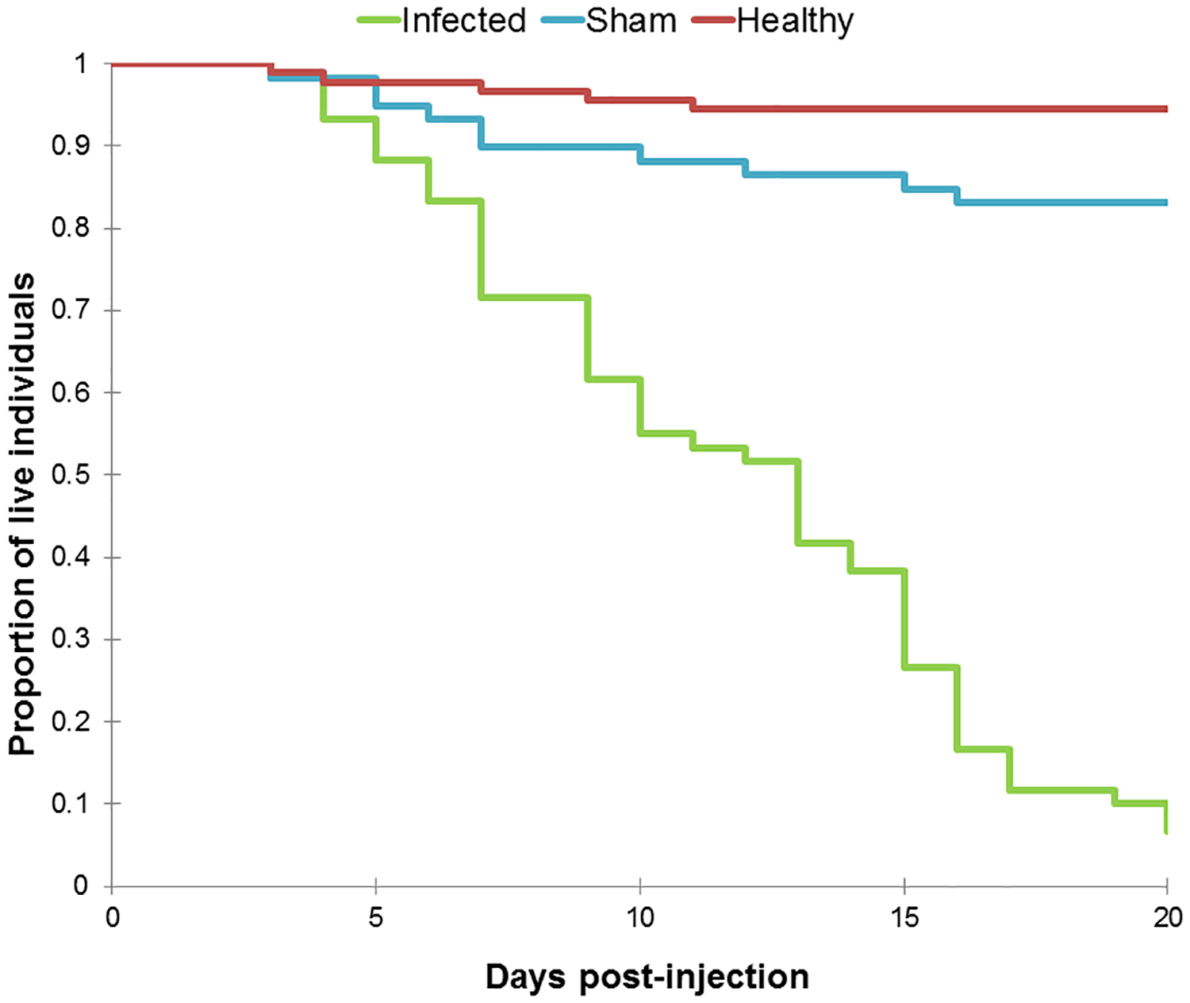
Survival rates. Each line color represents the survival rate observed in a different treatment: green (infected, n = 60), blue (sham, n = 59), and red (healthy, n = 91). Values on the y-axis denote the proportion of individuals which survived, while the x-axis represents time post-injection. We found individuals injected with *Ophiocordyceps kimflemingiae* had a significantly higher mortality rate than other treatments (Kaplan-Meier log rank: P<0.001).

**Fig 2:**
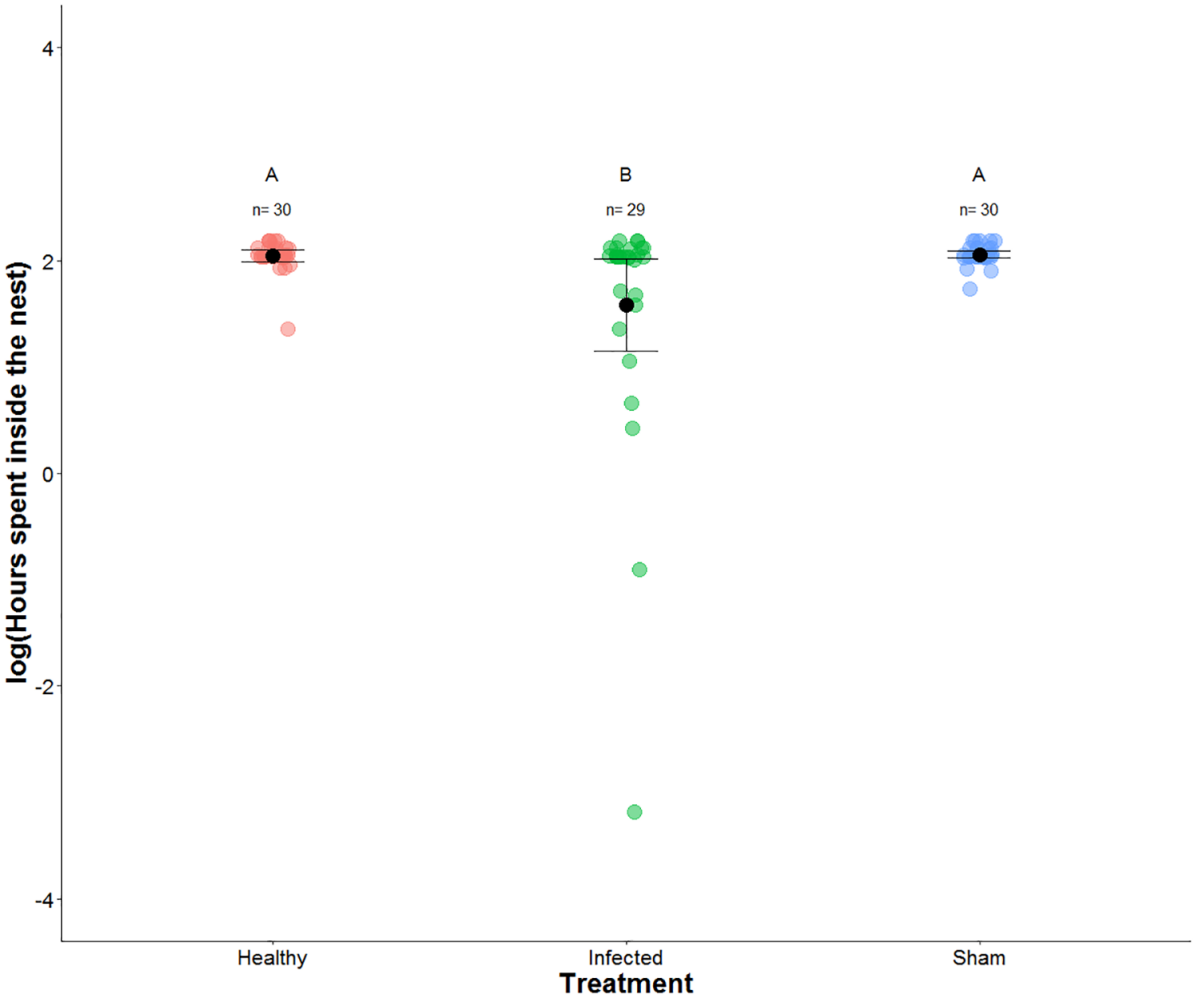
Time spent inside the nest 6 days post-injection. Each circle depicts the amount of time a focal individual spent inside the nest on day 6 post-injection. Each color represents a different treatment: red (healthy), blue (sham), and green (infected). The whisker plots depict the mean ± standard error for each treatment group, while the letters above the sample sizes denote significant differences between the treatments. Infected individuals spent less time inside the nest (GLMM: P<0.05).

Our study was not set up to monitor the end point of infection where the parasite controls the behavior resulting in the characteristic biting behavior. Instead we focused on the behavior of infected individuals inside the nest in the early stages of infection. We were unable to obtain proper *Ophiocordyceps kimflemingiae* growth *post mortem*. The reason for this was an abundance of environmental fungus from the genus *Aspergillus* which quickly colonized the cadaver. We are confident these fungi are not parasitic as no records exist of them infecting ants [20]. However, we are confident that the injection with *Ophiocordyceps kimflemingiae* resulted in established infections since these individuals died at a significantly higher rate than healthy or sham treatments (Fig 1; Kaplan-Meier log rank: P<0.001), independently of their colony or sub-colony. The low mortality (<20%) seen in sham treated individuals (those injected with only growth media) suggests that injection was not a cause of mortality in our observations. Another line of evidence supporting the assertion that those ants injected with *O*. *kimflemingiae* were infected was the amount of time such individuals spent inside of the nest. Previous observations by de Bekker et al. [14] saw infected workers leaving the nest 9 days after injection, and we saw similar behaviors (S1 Fig and S1 Table; ANOVA: F_2,18_ = 223, P<0.001). When taking into account the amount of time focal individuals spent inside the nest 6 days post-injection, we found infected individuals spent less time inside the nest compared to healthy (GLMM: P = 0.025) and sham (GLMM: P = 0.021) treatments (Fig 2).

### Infected ants are not attacked by siblings

In watching 585 hours of video footage, we saw no aggression towards infected ants by healthy ants or sham treated ants. Likewise we observed no aggression towards sham treated ants from their healthy siblings. The infected ants displayed no aggression.

### Infected ants engaged in trophallaxis with siblings

**Fig 3:**
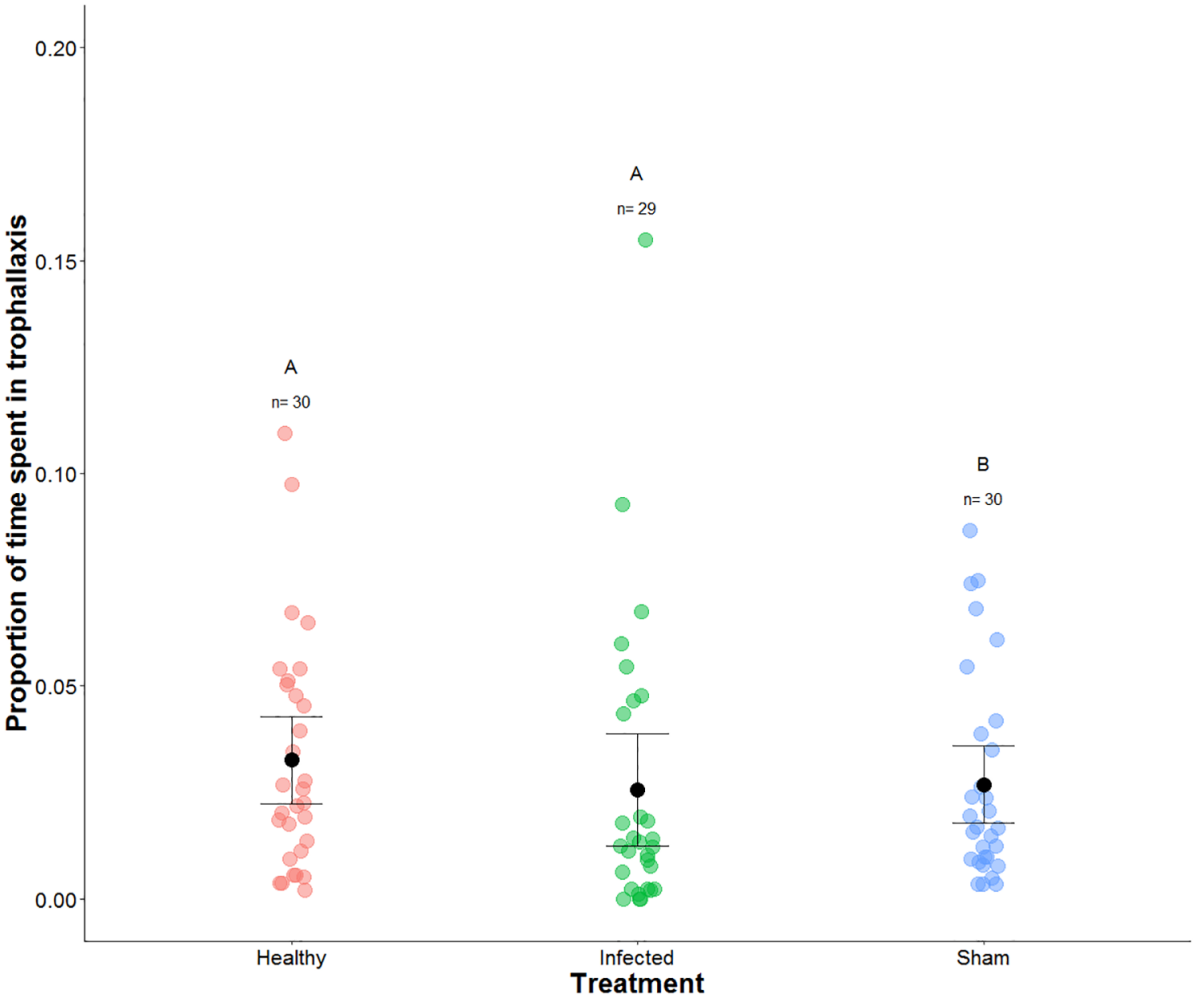
Proportion of time spent in trophallaxis 6 days post-injection. Each circle depicts the proportion of time a focal individual spent in trophallaxis on day 6 post-injection. The treatments are represented with different colors: red represents healthy (n = 30), green represents infected (n = 29), and blue represents sham (n = 30). The whisker plots depict the mean ± standard error for each treatment group, while the letters above the sample sizes denote significant differences between the treatments. Individuals within the sham treatment spent significantly less time in trophallaxis in comparison to the healthy treatment (GLMM: P = 0.034). The mixed-effect model used, along with the results can be found in Table 2.

Infected individuals received colony resources over the course of parasite development as determined by the frequency of trophallaxis. When comparing the trophallaxis rates from day 6 post injection, we found no significant difference between infected and uninfected treatments. However, individuals in the sham treatment spent significantly less time in trophallaxis compared to individuals in the healthy treatment (Fig 3 and Table 2; GLMM: P = 0.034). We performed the same analysis after removing the outlier found in the infected treatment (proportion of time spent in trophallaxis = 0.155) and arrived at similar results; the sham treatment was significantly different to the healthy treatment (GLMM: P = 0.033).

**Table 2:** Results from mixed-effect model on trophallaxis 6 days post-injection.

	P-values For Contrasts			Beta-hat values		
Model	H -I	I—S	H—S	Infected	Sham	Healthy
Square root (Proportion of time spent in trophallaxis)~ 0 + Treatment + (1|Colony.sub-colony) + (1|Ant identification)	0.067	0.330	0.034	0.126	0.143	0.160

Since trophallaxis involves two workers our data violates the assumption of independence. In order to correct for this we used a permutation test which permuted the treatment of each focal individual 10,000 times to obtain a dataset for comparison. We used the mixed-effect model denoted within this table to analyze our dataset. Letters differentiate amongst treatments: “H” for the healthy, “I” for the infected, and “S” for the sham treatment. When contrasting the treatments, we found individuals within the sham treatment are significantly different from the healthy treatment (GLMM: P<0.05).

### Infected ants are closer to the nest entrance

**Fig 4:**
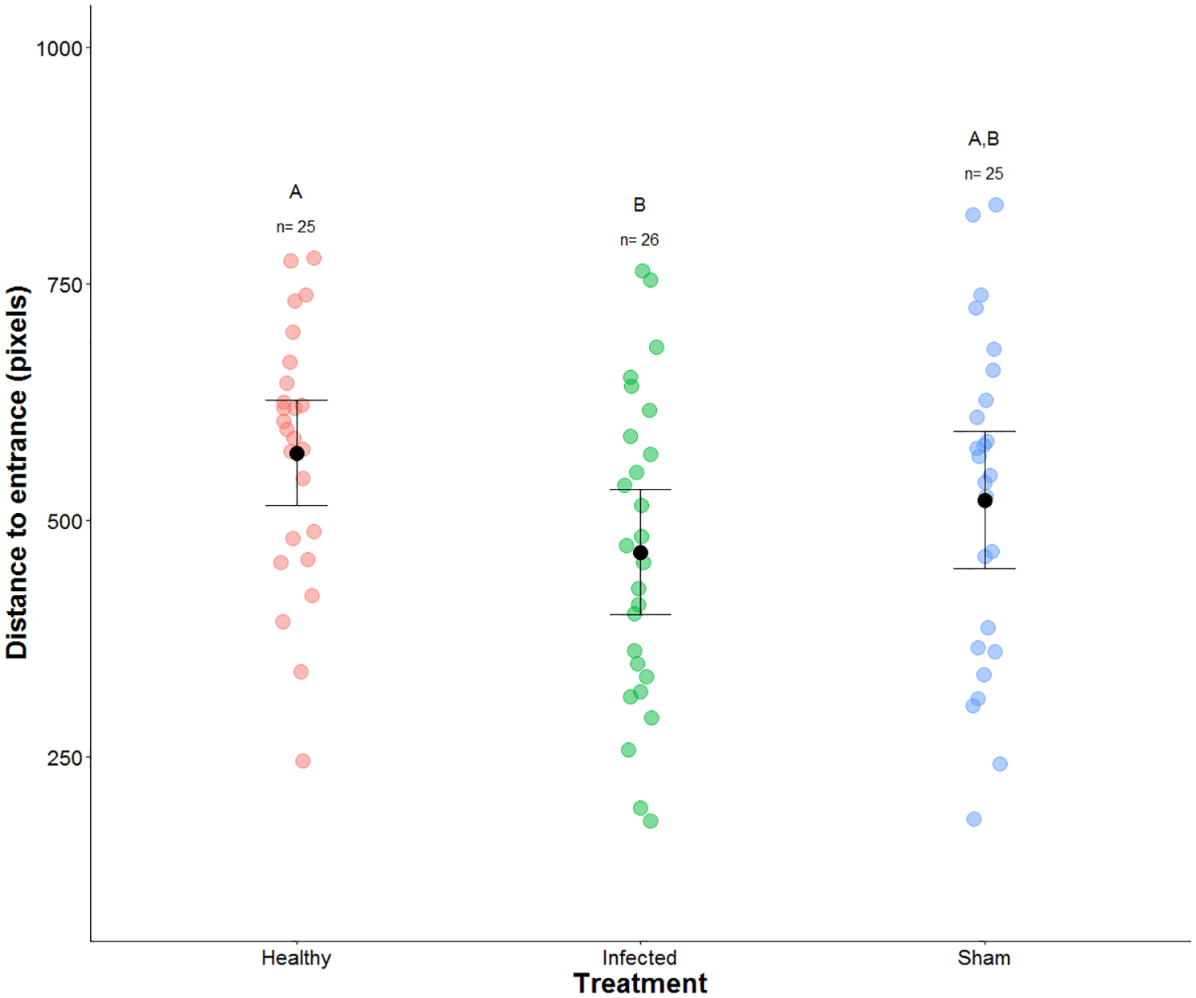
Mean distance between focal individuals and nest entrance. Each colored circle represents mean distance between a focal individual and the nest entrance 6 days post-injection. The treatments are represented with different colors: red represents healthy (n = 25), green represents infected (n = 26), and blue represents sham (n = 25). Above each group we used a letter to denote the significant differences between treatments, while the whisker plots depict the mean ± standard error for each treatment. We found infected individuals spent significantly more time closer to the nest entrance than the healthy treatment (GLMM: P = 0.023) The mixed-effect models, along with the results can be found on Table 3.

We used the worker’s location relative to the nest entrance as a proxy for spatial isolation, since it can be reasonably assumed that individuals near the nest entrance are isolated from the rest of the group. Infected individuals spent more time near the nest entrance in comparison to individuals in the healthy treatment (GLMM: P = 0.023; Fig 4 and Table 3). We found no significant difference between sham and the other treatments (GLMM: P>0.05).

**Table 3:** Analysis for the proximity of individuals and the entrance of the nest on day 6 post-injection.

	P-values for contrasts			Beta-hat values		
Model	H -I	I—S	H—S	Infected	Sham	Healthy
Distance to Entrance~ 0 + Treatment + (1|Colony.sub-colony) + (1|Ant identification)	0.023	0.252	0.294	468.735	521.853	571.535

We randomized the treatments of all focal individual 10,000 times before obtaining results from our mixed-effect model (depicted in table). In the contrasts section we used letters differentiate amongst treatments: “H” signifies the healthy, “I” for infected treatment, and “S” for sham treatment. We found infected individuals were significantly closer to the nest entrance in comparison to individuals within the healthy treatment (GLMM: P<0.05).

## Discussion

Our experiment set out to answer if ants infected by a specialized parasite would: 1) be recognized and attacked by their nestmates, 2) spend less of their time socially exchanging food than their uninfected peers, and 3) become spatially isolated inside of the nest. We hypothesized that infected individuals would be recognized and attacked, decreasing their rate of social food exchange. Furthermore, we expected that any increased aggression would lead to infected individuals becoming spatially isolated inside of the nest. In order to test these hypotheses, we observed the interactions between healthy *Camponotus castaneus* workers and those infected with *Ophiocordyceps kimflemingiae* in a sub-colony framework. We did not separate workers from their nestmates, we also allowed them to move freely between the nest and sandy foraging arena.

Based on 585 hours of observation we saw no attacks towards individuals injected with *O*. *kimflemingiae*. Our findings are similar to those in the Leclerc and Detrain [21] observations, where workers infected with *Metarhizium anisopliae* did not receive agnostic behaviors when placed in a nest context. However, observation in other social insect-parasite systems suggests that nest mates increase their aggression towards infected individuals. Baracchi et al. [22] demonstrated that uninfected honey bees (*Apis mellifera*) can recognize and increase their aggression towards individuals infected with Deformed Wing Virus (DWV). While termites increase their aggression to the point of cannibalism and burial of those that are ill. First noted by Fujii [23], in termites infected with entomopathogenic nematodes, infected workers would be cannibalized by their uninfected counter parts (“antenna and legs of moribund termites bitten off by nestmates”). Such behaviors have also been observed in scenarios using a generalist fungal entomopathogen, *Metarhizium anisopliae*, individuals exposed to conidia (asexual spores) would be attacked and in some occasions be buried by their unexposed counterparts [24]. However, many of these observations have been made using staged encounters, where workers are placed in a sterile environment and observed outside of the important social context of a nest. We propose that aggression studies performed on social insects should be based on observations within a biologically relevant social context similar to those performed here.

Aggression is not the only metric we can use to understand how individuals within the nest are treated. Observable changes in trophallaxis rates can inform us of the potential changes in social dynamics occurring inside of the nest, notably the exchange of food resources. However, note that the duration of trophallaxis cannot be considered an accurate measure of food intake, as ants constantly share the contents of their crop with other ants via trophallaxis, as well as exchange other resources (i.e. molecules with anti-biotic properties) [25, 26]. When we refer to trophallaxis we must acknowledge that it does not solely pertain to social food exchange, but also includes the exchange of other important resources amongst workers. We hypothesized infected individuals would decrease their rate of trophallaxis due to higher levels of aggression or because healthy individuals reduced the exchange of resources with infected ants. It would be beneficial for the colony to prevent infected individuals being fed, since these individuals will later become a source of disease to the colony [15].

Unlike other experiments and parasite systems [21, 26–31], we found sham individuals engaged in significantly less trophallaxis than individuals in the healthy and infected treatments (Fig 3 and Table 2). Workers of the species *Temnothorax nylanderi* infected with *Anomotaenia brevis* have been shown to increase trophallaxis and begging behaviors [30, 31]. Moreover de Souza et al. [27], Hamilton et al. [26], Aubert and Richard [28], and Qiu et al. [29] found affected workers increased trophallaxis after being exposed to infectious material or its components. However, many of these systems are comprised of infections with parasites or pathogens that have not coevolved with its host; in these examples there is only one coevolved system (*T*. *nylanderi* infected by *A*. *brevis*). Furthermore, the collection of trophallaxis observations within de Souza et al. [27], Hamilton et al. [26], Aubert and Richard [28], and Qiu et al. [29] are all staged interactions: individuals are placed within a petri dish and observed for short periods of time (ranging from 5 to 10 minutes). Dyad encounters could lead to bias conclusions, since workers are outside of the social network of the nest. Leclerc and Detrain [21] found that individuals infected with *M*. *anisopliae* encountered more challenges in dyadic encounters, but did not receive such challenges while inside the nest. Infected workers inside the nest were not actively rejected and received food over the course of fungal development [21], similar to our findings. In our experiment we use a more biologically relevant approach, workers are placed in a nest environment, in which workers were not separated from their nestmates, and are living within a nest.

The exclusion from socially shared food may be one of two ways workers can isolate infected individuals from other colony members. Infected individuals could be spatially isolated within the nest to reduce interactions or even encourages infected workers to leave the nest. Previous studies performed in the absence of parasites have shown workers have spatial fidelity while inside of the nest [32–34]. Here we have added the complexity of parasites, giving us an insight into how ant societies interact with their infected siblings. Previous work has noted that individuals infected with generalist entomopathogenic fungi, *Metarhizium brunneum* and *M*. *anisopliae*, spent significantly more time outside of the nest [35–37]. Suggesting that infected workers could be spatially isolated inside of the nest. We found that individuals infected with *O*. *kimflemingiae* spent significantly more time closer to the nest entrance in comparison to healthy individuals (Fig 4 and Table 3). However, we also found that sham individuals spent a significant amount of time in close proximity to the nest entrance. Findings which indicate that the treatment could have an effect on where ants place themselves inside the nest.

The extensive time infected individuals spent outside of the nest (Fig 2) and the results of our spatial analysis suggest infected individuals have different spatial fidelity compared to healthy colony members. However, small sample sizes may have an effect on our results. Since the conclusions made in our experiment could be dictated by individual variations, yet these extensive observations have given us insight into how infection is managed inside of an ant nest. We recommend future experiments to have more within and between colony replicates. Our results may reflect internal changes in the ant driven by the parasite which ultimately requires its host to leave the nest for onward transmission to occur [7, 10, 15, 16]. Similar observations have been previously made in other systems where ants were infected by generalist pathogenic fungi [21, 35–38].

## Conclusions

Within this experiment, we tested the response of a carpenter ant colony to the presence of infected siblings, which harbored the specialized fungal parasite *O*. *kimflemingiae*. We found infected individuals were not attacked, not excluded from colony resources, and spent significantly more time closer to the nest entrance in comparison to healthy workers. When considering the significance of these results, we must consider the biology of both the parasite and its host. Only individuals that leave the nest to collect food are infected by *O*. *kimflemingiae* and the infection cannot be transmitted within the nest [15]. Foragers only account for a small portion of the colony’s population, roughly 30%, and likely only a small portion of foragers get infected [39]. The costs of parasitism on the colony are low and can be buffered if the colony is mature enough [40]. Therefore, *O*. *kimflemingiae* infection may not necessarily exert a strong selection for the evolution of behaviors which would inhibit parasite development, such as aggression, exclusion, and isolation. Taking into account our observations and the biology of *O*. *kimflemingiae*, we suggest *O*. *kimflemingiae* is a chronic parasite of the colony that is able to survive without triggering strong behavioral defenses in the society, in short the parasite is able to fly under the radar of the colony’s defenses.

## Supporting information

S1 Fig Time spent within the nest. Each line represents the proportion of time each treatment spent inside the nest: red (healthy), blue (sham), and green (infected). We performed observations over the course of seven days (3–9 days post-injection), during the daylight hours (0900–1700). Infected individuals spent overall less time inside the nest (ANOVA: F_2,18_ = 223, P<0.001). The sample sizes for these data can be seen in S1 Table.
(TIF)

S1 Table Sample sizes for percent time spent within the nest. We performed the observations over the course of seven days (3–9 days post-injection), during the daylight hours (0900–1700). We collected these data from both sub-colonies of genetic colony 3. Each cell has the number of focal individuals followed per day to determine the percent of time each treatment spent inside the nest.
(PDF)

S1 File Mortality data. We performed daily mortality observations for up to 20 days post-injection. In this table we present the raw data we used for analysis. Individuals which died over the course of our observations had a 1 death status.
(CSV)

S2 File Trophallaxis and the amount of time spent inside the nest. We followed each focal individual 6 days post-injection to determine the amount of time spent inside the nest and how long they spent exchanging food socially. In this table we present the raw data used for analysis.
(CSV)

S3 File Spatial data. We measured the distance between each focal individual and the nest entrance every minute from 0900–1700. In this table we present the raw data used for analysis. Distances with NA symbolize periods for which workers were outside of the nest.
(CSV)
