# Supplementary figures and images for: Within the fortress: A specialized parasite is not discriminated against in a social insect society
Source: PLoS One. 2018 Feb 23;13(2):e0193536. doi: 10.1371/journal.pone.0193536 (PMC5825133; doi:10.1371/journal.pone.0193536)

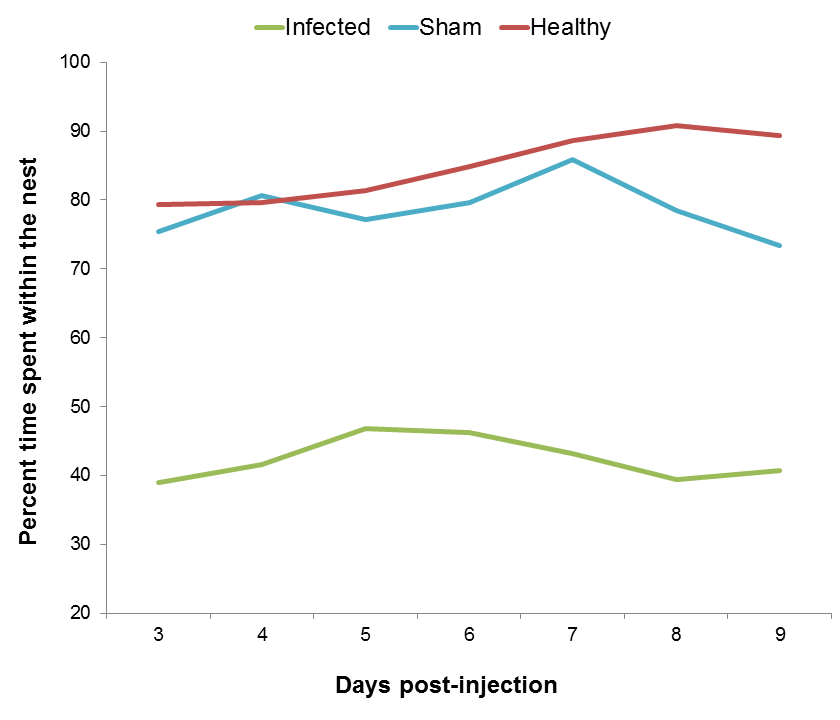

Supplement: S1 Fig — Each line represents the proportion of time each treatment spent inside the nest: red (healthy), blue (sham), and green (infected). We performed observations over the course of seven days (3–9 days post-injection), during the daylight hours (0900–1700). Infected individuals spent overall less time inside the nest (ANOVA: F2,18 = 223, P<0.001). The sample sizes for these data can be seen in S1 Table. (TIF) [file pone.0193536.s001.TIF]
