# Supplementary material for: Within the fortress: A specialized parasite is not discriminated against in a social insect society
Source: PLoS One. 2018 Feb 23;13(2):e0193536. doi: 10.1371/journal.pone.0193536 (PMC5825133; doi:10.1371/journal.pone.0193536)
Supplement: S1 Table — We performed the observations over the course of seven days (3–9 days post-injection), during the daylight hours (0900–1700). We collected these data from both sub-colonies of genetic colony 3. Each cell has the number of focal individuals followed per day to determine the percent of time each treatment spent inside the nest. (PDF) [file pone.0193536.s002.pdf]

S1 Table. Sample sizes for percent time spent within the nest.

	**Day post-injection**						
**Treatment**	**3**	**4**	**5**	**6**	**7**	**8**	**9**
Infected	20	19	19	18	17	15	13
Sham	20	20	19	19	19	19	19
Healthy	31	30	29	29	28	28	28

We performed the observations over the course of seven days (3-9 days post-injection), during the daylight hours (0900-1700). We collected these data from both sub-colonies of genetic colony 3. Each cell has the number of focal individuals followed per day to determine the percent of time each treatment spent inside the nest.
